# Myeloperoxidase expressing tumor associated neutrophils are associated with worse prognosis in metastatic breast cancer patients

**DOI:** 10.1038/s41598-025-08854-x

**Published:** 2025-07-12

**Authors:** Karin Leandersson, Dag Blomgård, Oscar Tuvesson, Lisa Rydén, Caroline Bergenfelz, Anna-Maria Larsson

**Affiliations:** 1 Cancer Immunology, Department of Translational Medicine, Lund University, Malmö, Sweden; 2 Division of Experimental Infection Medicine, Department of Translational Medicine, Lund University, Malmö, Sweden; 3 Division of Oncology, Department of Clinical Sciences Lund, Lund University, Lund, Sweden; 4 Department of Surgery and Gastroenterology, Skåne University Hospital, Malmö, Sweden

**Keywords:** Metastatic breast cancer (MBC), TAN, TAM, MPO, G-MDSC, Breast cancer, Tumour biomarkers, Tumour immunology, Breast cancer

## Abstract

Tumor associated neutrophils (TANs) exert dual and opposing functions in tumors, acting pro-tumorigenic and anti-tumorigenic, depending on tumor progression, polarization state and subtype. Consequently, the prognostic impact of TANs in breast cancer is also contradictory. Since neutrophils are critically needed to fight infections in cancer patients, the mediators leading to tumor progression need more investigation as potential future targets. The neutrophil derived mediator myeloperoxidase (MPO) is a peroxidase with dual functions in tumors, acting both immune enhancing and suppressing. Patients with metastatic breast cancer (MBC) have aggressive tumors with a dismal prognosis and urgently need novel treatment strategies. Therefore, we here aimed to investigate the prognostic impact of TANs, MPO^+^ TANs and MPO^+^ non-neutrophils using a cohort with newly diagnosed MBC patients specifically. We show that high infiltration of MPO^+^ TANs and MPO^+^ non-neutrophils in the primary tumor (PT), was associated with clinicopathological features and worse prognosis in patients with MBC. However, only infiltration of MPO^+^ TANs showed independent prognostic impact in multivariable analysis adjusting for other prognostic factors in MBC. The results need to be validated in a larger cohort but suggests that MPO targeting strategies could be relevant in breast cancer patients with aggressive disease.

**Supplementary Information:**

The online version contains supplementary material available at 10.1038/s41598-025-08854-x.

## Introduction

Breast cancer is the most common type of cancer in women and the leading cause of cancer related deaths among women worldwide^1^. There are different molecular subtypes of breast cancer, defined by hormone receptor expression status (Estrogen receptor; ER, and Progesterone receptor; PR) and Human epidermal growth factor receptor 2 (HER2) status, affecting prognosis^2,3^. The novel immune checkpoint inhibitors (ICI) modifying the adaptive anti-tumor immune response is relatively ineffective in breast cancer, with a possible exception for triple negative breast cancer (TNBC)^4,5^. Therefore, knowledge regarding the innate immune cell populations infiltrating the tumor microenvironment (TME) need further investigation for us to understand and improve the efficacy of current treatments^6,7^ and to develop novel therapies.

The innate myeloid immune cells are the most common immune cells in the TME compartment^8^ and are crucial for activation of the adaptive immune response against tumors. Nevertheless, infiltration of innate myeloid immune cells in tumors, are generally associated with a worse outcome for cancer patients, promoting immune evasion^9,10^. The prognostic effect of tumor associated neutrophils (TANs) is however still debated^10,11^ also in breast cancer patients^12–15^. These contradictory findings may be caused by different patient and tumor characteristics in the cohorts analyzed, or by the dual nature of neutrophils, being able to act in both anti-tumorigenic (N1 neutrophils) and pro-tumorigenic (N2 type neutrophils) manners depending on tumor stage and polarization state^9–13,16^. N1 neutrophils have a short lifespan and cause tumor cell death via direct mechanisms including degranulation, respiratory burst and reactive oxygen species (ROS) derived from neutrophil extracellular traps (NETs)^17^. In contrast, N2 neutrophils can survive for longer time and have been proposed to play important roles in orchestrating inflammatory responses, angiogenesis, tumor promoting cell death-dependent neutrophil extracellular trap formation (NETosis) and immunosuppression leading to tumor progression^10,11,18^. N2 neutrophils are related, or perhaps even identical, to the strongly immunosuppressive granulocytic myeloid suppressor cells (G-MDSCs or PMN-MDSCs)^19–23^ that also have a prolonged life-span^24,25^. We have previously reported that G-MDSCs in patients with metastatic breast cancer (MBC) represent a heterogeneous population of cells from the neutrophil lineage, where the majority represents mature neutrophils most likely representing N2 neutrophils^26^.

Myeloperoxidase (MPO) is a lysosomal peroxidase released by neutrophils during degranulation. It acts antimicrobial^27^ and is required for the formation of NETs^28,29^. It is primarily expressed by mature neutrophils and occasionally in monocytes, with expression of MPO usually disappearing upon differentiation into macrophages^28,30^. MPO can therefore also serve as a maturation and activation marker for neutrophils. Besides the oxidative effects, it can also affect nitrosylation, immunomodulation, cause tissue damage but also promote tissue remodeling and repair^27^. Cells expressing MPO have previously been shown to infiltrate tumors, with various prognostic impact^30^. In breast cancer patients an increased serum-MPO level was shown, and the risk of developing breast cancer increased with higher endogenous levels of MPO^31^. Furthermore, MPO has been shown to promote progression of breast cancer both in vitro and in an in vivo model^30^. It has previously been reported that MPO-expressing cells in breast tumors are an independent positive prognostic marker^32^. Whether these cells were neutrophils or other cell types were not concluded. However, we recently showed that MPO^+^ neutrophils expressing the transcription factor Autoimmune regulator (AIRE) in breast tumors were associated with worse prognosis in breast cancer patients^33^ although only MPO-expressing neutrophils were not prognostic using the same cohort^26^.

Lately, novel immune targeting strategies focusing on TANs and MPO have been proposed^12,13,30^. Elucidating the role for TANs in a cohort of patients with aggressive breast cancer that potentially would be eligible for future immunotherapy options targeting neutrophil functions (i.e. MBC patients) is therefore important.

Here, we aimed to investigate the potential prognostic relevance of TANs, MPO-expressing TANs and non-neutrophils in primary tumors (PT), from a cohort of newly diagnosed MBC patients in an observational study with long follow-up.

## Results

### Patient and tumor characteristics

**Fig. 1:**
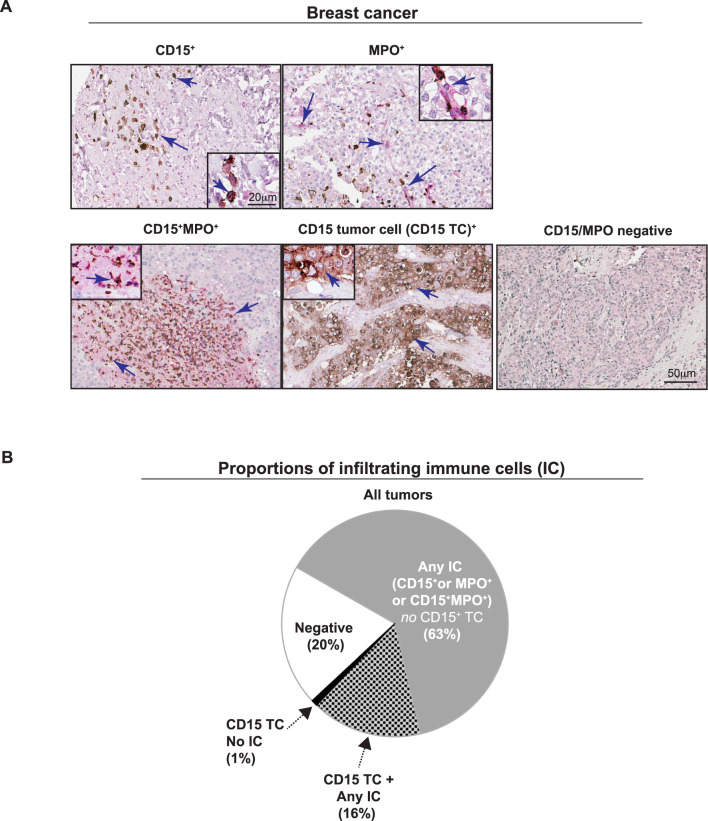
Immunohistochemical (IHC) analyses of MPO^+^ TANs and MPO^+^ non-neutrophils in PT from MBC patients. (**A**) IHC double staining of CD15 (brown) and MPO (pink) in human breast cancer tissue, where CD15^+^MPO^−^ cells represent TANs/G-MDSCs in an immature/non-activated state, CD15^+^MPO^+^ mature/activated TANs/G-MDSCs, CD15^−^MPO^+^ non-neutrophils (monocyte/macrophages) expressing MPO and CD15^+^ TCs malignant cells expressing CD15. All histological sections were counterstained with H&E. The scale bars shown are representative for all images (20 μm for magnified inserts and 50 μm for full images). (**B**) Pie chart showing descriptive statistics of amount (%) or tumors with infiltration of any immune cell (IC; grey) (CD15^+^MPO^−^, CD15^−^MPO^+^, CD15^+^MPO^+^), CD15^+^ TCs with simultaneous infiltration of any IC (checkered), CD15^+^ TCs alone (black) or no staining (negative; white). Also see Supplementary Table 2.

Patient and tumor characteristics of the 156 patients with newly diagnosed MBC in the original cohort have been published before^34–36^. Of these 156 patients, PT tissue samples were available from 114 patients and scored for CD15 and MPO expression (Fig. 1). Patient and tumor characteristics of the CD15/MPO cohort, compared to the original cohort, are summarized in Supplementary Table 1. Considering MBC subtype (based on metastases first-hand and PT secondly), 78 patients (72%) had ER-positive (HER2-negative) disease, 14 (13%) had HER2-positive disease and 17 (16%) had TNBC disease as determined by ER and HER2 expression. Regarding molecular subtype using PAM50 subtyping of PT, 45 patients (40%) had Luminal A, 39 patients (35%) Luminal B, 15 patients (13%) had HER2-enriched, and 14 patients (12%) had basal-like PT. Regarding the metastatic disease, 23 patients (20%) had *de novo* MBC (metastatic disease at initial diagnosis), whereas 20 patients (18%) had a metastasis-free interval (MFI) of ≤ 3 years and 71 patients (62%) had a MFI of > 3 years. Considering number of metastatic sites, 39 patients (34%) had ≥ 3 metastatic sites whereas 75 patients (66%) had < 3 metastatic sites. Furthermore, 68 patients (60%) had visceral metastases (metastases in ascites, pleura, liver, lungs or the central nervous system) at time of MBC diagnosis. Patients were included before start of systemic therapy for MBC and received treatment based on national clinical guidelines determined by the treating physician. 54 patients (50%) received chemotherapy as first line systemic therapy after study inclusion; 43 patients (40%) received endocrine therapy and 10 patients (9%) HER-2 directed therapy (Supplementary Table 1). Patients were followed-up every three months with radiological and clinical evaluation as previously described^34^. The median follow-up time was 97 months (75–123 months).

### Presence of CD15^+^and MPO^+^ myeloid cells in primary tumors (PT) of MBC patients

In this study, all IHC was performed on the PT of MBC patients. We annotated the staining according to cells expressing CD15^+^MPO^−^ (CD15^+^; immature or non-activated neutrophils (brown only), CD15^−^MPO^+^ (MPO^+^; any cell expressing MPO other than neutrophil (pink only)), CD15^+^MPO^+^ (mature and activated neutrophils expressing MPO (brown and pink co-staining)) expression in immune cells, or CD15^+^ expression in malignant tumor cells (CD15^+^ TC) (Fig. 1A). MPO expression in malignant cells was not found and hence not presented. The majority of CD15^+^ neutrophils in the primary breast tumors of MBC patients were mature/activated TANs/G-MDSCs. 49% of tumors were infiltrated by mature/activated TANs/G-MDSCs (CD15^+^MPO^+^ cells; 56 out of 114 tumors); 39% of tumors were infiltrated by immature/non-activated TANs/G-MDSCs (CD15^+^MPO^−^ cells; 41 out of 104 tumors; Fig. 1B and Supplementary Table 2). In 10 cores, the CD15^+^MPO^−^ staining could not be confidently identified as belonging to an immune cell (IC) or other cell type and was therefore not scored. With regards to MPO expression alone, 65% of tumors were infiltrated by MPO expressing cells of non-neutrophil origin (CD15^−^ MPO^+^ cells; 74 out of 114 tumors; Fig. 1B and Supplementary Table 2) and out of the tumors that had presence of MPO expressing cells of non-neutrophil origin (CD15^−^ MPO^+^ cells), 41% also had simultaneous presence of MPO expressing cells of TAN/G-MDSC origin (CD15^+^MPO^+^ cells; 47 out of 114 tumors; Fig. 1B and Supplementary Table 2). For tumors with infiltration of immature/non-activated TANs/G-MDSCs (CD15^+^MPO^−^ cells), 18% also had simultaneous presence of mature/activated MPO expressing cells of TAN/G-MDSC origin (CD15^+^MPO^+^ cells; 19 out of 104 tumors; Fig. 1B and Supplementary Table 2). 15 tumors (13%) had simultaneous infiltration of all IC annotated (CD15^+^MPO^+^ cells, CD15^+^MPO^−^ cells and CD15^−^ MPO^+^ cells; Fig. 1B and Supplementary Table 2). 17% of tumors had CD15^+^ expressed in tumor cells (CD15^+^ TCs) (19 of 114 tumors; Fig. 1B) and the majority had simultaneous infiltration of any IC (16%; Fig. 1B and Supplementary Table 2). The annotated ICs could be found both in the tumor parenchyma and stromal areas.

### MPO^+^ TANs associate with adverse clinicopathological features in MBC patients

To investigate the potential effect of TANs and MPO-expressing cells (CD15^+^MPO^+^ and CD15^−^MPO^+^) on breast tumor progression, we initially performed bivariate Spearman´s 2-tailed correlation analyses test in relation to clinicopathological features in the primary tumors (PT; NHG, tumor size, nodal stage, breast cancer subtype, Ki67) and age at PT or metastasis (Table 1). We found that while infiltration of CD15^+^MPO^+^ cells in PT correlated with NHG and Ki67, the presence of CD15^−^MPO^+^ cells correlated significantly with NHG, Ki67 and tumor size. Both CD15^−^MPO^+^ and CD15^+^MPO^+^ cells also correlated with presence of stromal tumor infiltrating lymphocytes (TILs). CD15^+^MPO^−^ cells and CD15^+^ TC were not associated with any parameter tested (Table 1).

**Table 1:** Correlations^a^ between clinicopathological features of the primary breast tumor (PT) and presence of CD15^+^, MPO^+^, CD15^+^/MPO^+^ immune cells, or CD15^+^ tumor cells (CD15^+^ TC) in PT.

Clinicopathological features	CD15^+^			MPO^+^			CD15^+^MPO^+^			CD15^+^ TC		
	Correlation Coefficient	*P* value (2-tailed)	N	Correlation Coefficient	*P* value (2-tailed)	N	Correlation Coefficient	*P* value (2-tailed)	N	Correlation Coefficient	*P* value (2-tailed)	N
Age at PT Age at Met NHG Tumor size Nodal stage Breast cancer subtype^b^ Ki67 Stromal TILs	0.068 0.026 − 0.159 0.004 0.050 − 0.028 − 0.003 0.095	0.492 0.791 0.157 0.969 0.643 0.798 0.975 0.365	104 104 81 98 89 85 100 92	0.015 0.067 **0.379** **− 0.312** -0.129 0.037 **0.206** **0.418**	0.875 0.476 <0.001*** 0.001** 0.203 0.723 0.032* <0.001***	114 114 90 108 99 93 109 101	0.052 0.035 **0.347** − 0.077 − 0.133 0.075 **0.211** **0.237**	0.585 0.709 <0.001*** 0.431 0.189 0.475 0.027* 0.017*	114 114 90 108 99 93 109 101	0.080 0.033 0.132 − 0.020 0.054 0.034 − 0.073 -0.077	0.397 0.730 0.215 0.835 0.596 0.743 0.451 0.446	114 114 90 108 99 93 109 101

^a^ Spearman’s 2-tailed test * *P* < 0.05, ** *P* < 0.01, *** *P* < 0.001. NHG = Nottingham Histological grade, PT = primary tumor, Met = Metastasis.

^b^ Breast cancer subtype was primarily derived from immunohistochemical staining.

In summary, CD15^+^MPO^+^ cells and MPO^+^ TANs present in PT associate with adverse clinicopathological features and stromal TILs in MBC patients.

### MPO^+^ neutrophils are associated with a worse prognosis in MBC patients

**Fig. 2:**
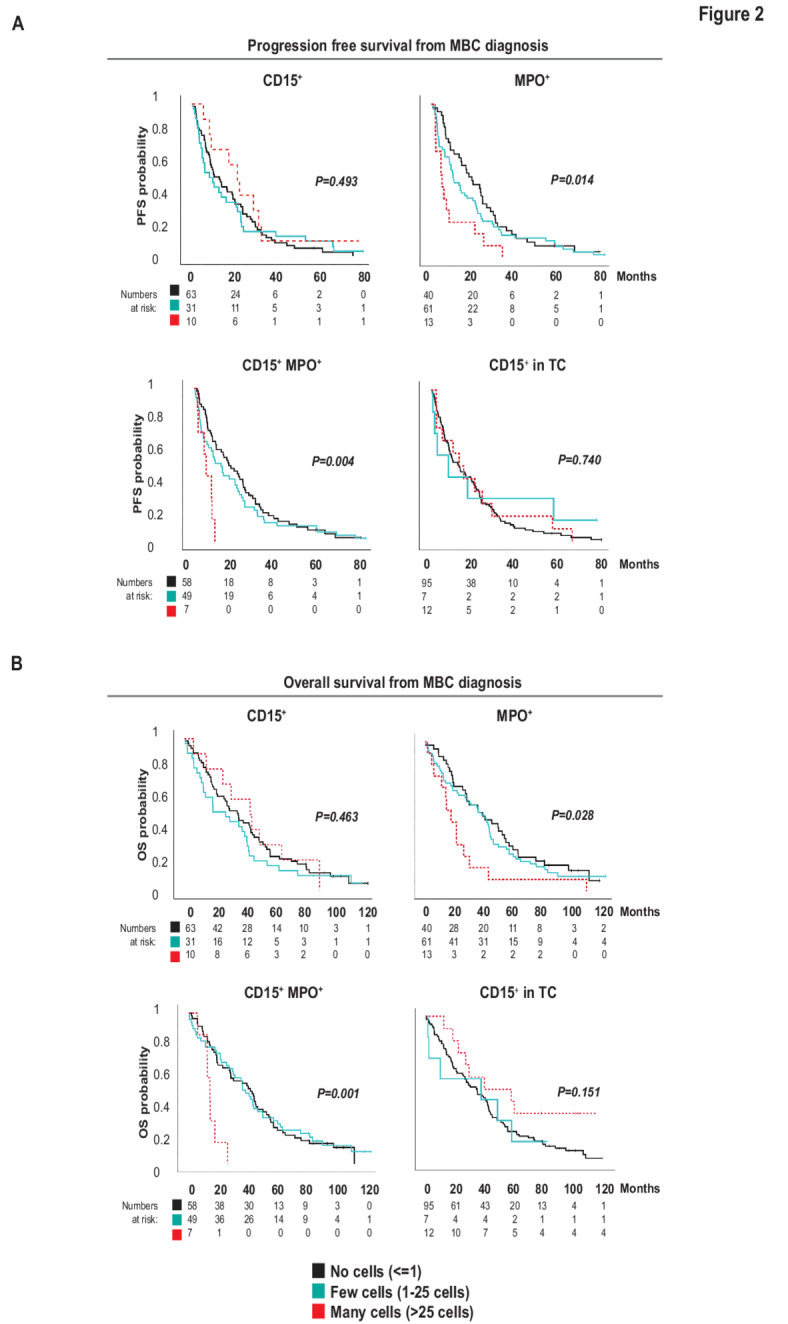
Presence of MPO^+^ TANs is associated with worse prognosis in MBC patients. (**A**) Kaplan-Meier curves illustrating differences in progression-free survival (PFS) according to CD15 and MPO expression in MBC patients. The cores were annotated for presence of cells expressing the markers according to grading no cells ( < = 1 cells; black), few cells (1–25 cells; turquoise) or many cells (> 25 cells; red). Log-rank *P*-value < 0.05 was considered significant. (**B**) Kaplan-Meier curves illustrating differences in overall survival (OS) according to CD15 and MPO expression in MBC patients. The cores were annotated for presence of cells expressing the markers according to grading no cells ( < = 1 cells; black), few cells (1–25 cells; turquoise) or many cells (> 25 cells; red dashed line). Log-rank *P*-value < 0.05 was considered significant.

We next analyzed whether infiltration of TANs and MPO-expressing cells in the PT could impact progression free survival (PFS) and overall survival (OS) in MBC patients measured from time of MBC diagnosis. We graded scorings as the absence (no cells; <=1 cell), or presence of low infiltration (few cells; 1–25 cells) or high infiltration (many cells; >25 cells) of CD15^+^MPO^−^ cells, CD15^+^MPO^+^ cells, CD15^−^MPO^+^ cells and CD15^+^ TCs respectively (representative IHC shown in Fig. 1A). We found that in MBC patients, PFS and OS was significantly associated with previous PT infiltration of a higher number of CD15^−^MPO^+^ cells (MPO^+^) and of MPO^+^ TANs in particular (CD15^+^MPO^+^) (PFS; Fig. 2A) (MPO^+^; *P* = 0.014 and CD15^+^MPO^+^; *P* = 0.004) and (OS; Fig. 2B) (MPO^+^; *P* = 0.028 and CD15^+^MPO^+^; *P* = 0.001). This was not seen for cells expressing CD15^+^MPO^−^ (CD15^+^; non-activated or immature neutrophils; Fig. 2A-B) (PFS CD15^+^; *P* = 0.493 and OS CD15^+^ TC; *P* = 0.463), or CD15^+^ expression in malignant tumor cells (CD15^+^ TC; Fig. 2A-B) (PFS CD15^+^ TC; *P* = 0.740 and OS CD15^+^ TC; *P* = 0.151).

In summary, in MBC patients, the previous infiltration of MPO^+^ cells and of CD15^+^MPO^+^ TANs into the PT was associated with a shorter PFS and OS, measured in time from MBC inclusion to readout.

### Multivariable Cox regression analyses of MPO^+^ TANs in breast cancer

We next performed multivariate (MV) Cox regression analyses to investigate whether the TANs and MPO^+^ cells infiltrating the PT had an independent prognostic impact on MBC patient prognosis (Table 2). Analyses were adjusted for established prognostic factors in MBC including age, Eastern Cooperative Oncology group (ECOG) performance status, NHG, breast cancer subtype (ER^+^/HER2^−^, HER2^+^, TNBC), metastasis-free interval (MFI), number of metastatic sites (< 3 / >=3) and site of metastasis (visceral / non-visceral). Whereas unadjusted analyses of infiltration of many MPO^+^ (CD15^−^MPO^+^) cells (*P* = 0.004) and CD15^+^MPO^+^ TANs (*P* = 0.002) showed significant associations with PFS (Table 2), adjusted MV analyses indicated that only infiltration of many CD15^+^MPO^+^ TANs had independent prognostic impact on PFS (adjusted MV analyses PFS: CD15^−^MPO^+^ many cells; (*HR* = 1.65, 95%*CI*: (0.74–3.69), *P* = 0.22 and CD15^+^/MPO^+^; (*HR* = 3.04, 95%*CI*: (1.13–8.19), *P* = 0.028) (Table 2). Significant associations with a shorter OS were found in unadjusted analyses of infiltration of many MPO^+^ cells (*P* = 0.010) and CD15^+^/MPO^+^ TANs (*P* < 0.001) in the PT (Table 2). This did, however, not hold as independent factors after adjusting for other prognostic factors (adjusted MV analyses OS: CD15^−^MPO^+^; (*HR* = 1.33, 95%*CI*: (0.61–2.88), *P* = 0.480 and CD15^+^/MPO^+^; (*HR* = 2.04, 95%*CI*: (0.75–5.54), *P* = 0.160). In relation to PFS, unadjusted analyses for infiltration of many CD15^+^MPO^−^ TANs (*P* = 0.270) and presence of many CD15^+^ TCs (*P* = 0.800) in the PT, and in relation to OS for CD15^+^MPO^−^ TANs (*P* = 0.694) and CD15^+^ TCs (*P* = 0.057) (Table 2), were not significant. However after adjusting for prognostic factors in MV analyses, presence of many CD15^+^ TCs in the PT showed to have independent impact (adjusted MV analyses PFS: many CD15^+^MPO^−^ TANs; (*HR* = 0.58, 95%*CI*: (0.22–1.50), *P* = 0.260 and CD15^+^ TCs; (*HR* = 0.56, 95%*CI*: (0.26–1.21), *P* = 0.140) and adjusted MV analyses OS: many CD15^+^MPO^−^ TANs; (*HR* = 0.72, 95%*CI*: (0.26–1.98), *P* = 0.530 and CD15^+^ TCs; (*HR* = 0.30, 95%*CI*: (0.12–0.74), *P* = 0.009) (Table 2).

**Table 2:** Cox regression models on outcome by CD15^+^, MPO^+^, CD15^+^/MPO^+^ immune cells, or CD15^+^ tumor cells (CD15^+^ TC) in primary breast tumors.

Variable	Progression-free survival Hazard ratio (95% CI)				Overall survival Hazard ratio (95% CI)			
	Unadjusted	*P*-value	Adjusted	*P*-value	Unadjusted	*P*-value	Adjusted	*P*-value
**Primary tumor** CD15^+^ few cells CD15^+^ many cells MPO^+^ few cells MPO^+^ many cells CD15^+^MPO^+^ few cells CD15^+^MPO^+^ many cells CD15^+^ TC – few cells CD15^+^ TC – many cells	1.04 (0.67–1.63) 0.67 (0.33–1.36) 1.26 (0.83–1.90) 2.52 (1.33–4.78) 1.20 (0.81–1.77) 3.77 (1.65–8.60) 0.72 (0.31–1.68) 0.93 (0.51–1.70)	0.85 0.27 0.27 **0.004** 0.37 **0.002** 0.45 0.80	0.88 (0.51–1.55) 0.58 (0.22–1.50) 0.71 (0.39–1.3) 1.65 (0.74–3.69) 0.94 (0.55–1.60) 3.04 (1.13–8.19) 0.92 (0.34–2.52) 0.56 (0.26–1.21)	0.67 0.26 0.27 0.22 0.81 **0.028** 0.87 0.14	1.28 (0.81-2.00) 0.87 (0.43–1.76) 1.17 (0.76–1.79) 2.35 (1.23–4.49) 0.96 (0.64–1.45) 4.12 (1.78–9.57) 0.99 (0.43–2.28) 0.49 (0.24–1.02)	0.288 0.694 0.480 **0.010** 0.856 **< 0.001** 0.989 0.057	1.15 (0.66-2.00) 0.72 (0.26–1.98) 0.64 (0.36–1.14) 1.33 (0.61–2.88) 0.75 (0.43–1.31) 2.04 (0.75–5.54) 1.00 (0.36–2.85) 0.30 (0.12–0.74)	0.61 0.53 0.13 0.48 0.31 0.16 0.99 **0.009**

Adjusted for age at metastatic breast cancer diagnosis, ECOG performance status (0, 1, 2), NHG (III versus I-II), Subtype (ER+/HER2-, HER2+, TNBC), MFI (0, > 0–3, > 3), number of metastatic sites (< 3/>=3), site of metastasis (visceral/non-visceral).

Hence, in patients with MBC, former infiltration of CD15^+^MPO^+^ TANs in the PT showed to be an independent prognostic factor for PFS, while the presence of CD15^+^ TCs in the PT was an independent prognostic factor for OS, as measured in time from MBC diagnosis.

### Presence of MPO^+^ TANs associate with timing and site of distant metastasis

Given the worse prognosis for patients with high amounts of TANs or MPO^+^ cells, we next investigated whether presence of CD15^+^ TANs or MPO^+^ cells in PT would associate with timing or site of distant metastasis (Table 3). In agreement with having a worse survival, we could see that presence of CD15^+^MPO^+^ TANs or MPO^+^ cells in general, correlated significantly with recurrent distant metastasis (DM) when compared to *de novo* MBC (MBC at initial diagnosis) (MPO^+^ TANs: *P* = 0.001 and CD15^+^MPO^+^ cells: *P* = 0.001) (Table 3). CD15^+^MPO^−^ TANs also correlated inversely with skin metastasis (*P* = 0.020), a trend was seen for association between CD15^−^MPO^+^ (MPO^+^) cells and lung metastasis (*P* = 0.059; Table 3), while CD15^+^MPO^+^ TANs showed a negative trend towards correlating with bone metastasis (*P* = 0.061). Finally, CD15^+^ TCs showed a significant association to CNS metastasis (*P* = 0.038; Table 3).

**Table 3:** Correlations^a^ between site for distant metastasis (DM) in relation to immune cell score (variable), i.e., presence of CD15^+^, MPO^+^, CD15/MPO^+^ immune cells, or CD15^+^ tumor cells (CD15^+^ TC) in the primary breast tumor (PT).

Site of recurrence	CD15^+^			MPO^+^			CD15^+^MPO^+^			CD15^+^ TC		
	Correlation Coefficient	*P* value (2-tailed)	N	Correlation Coefficient	*P* value (2-tailed)	N	Correlation Coefficient	*P* value (2-tailed)	N	Correlation Coefficient	*P* value (2-tailed)	N
Recurrent DM vs. *de novo* MBC	0.031	0.756	104	**0.304**	0.001**	114	**0.301**	0.001**	114	0.057	0.544	114
DM Lymph nodes	− 0.036	0.719	104	0.005	0.959	114	0.083	0.380	114	0.008	0.937	114
DM Ascites	0.062	0.529	104	− 0.016	0.864	114	− 0.095	0.313	114	− 0.085	0.369	114
DM Pleura	− 0.150	0.130	104	0.074	0.437	114	− 0.040	0.669	114	− 0.010	0.917	114
DM Lung	− 0.014	0.885	104	0.177	**0.059**	114	0.041	0.664	114	− 0.076	0.422	114
DM Liver	0.125	0.206	104	0.025	0.790	114	− 0.025	0.791	114	− 0.057	0.549	114
DM Skin	**− 0.228**	0.020*	104	0.094	0.318	114	0.117	0.215	114	− 0.122	0.195	114
DM CNS	0.148	0.134	104	0.157	0.095	114	− 0.082	0.386	114	**0.194**	0.038*	114
DM Skeleton	0.124	0.210	104	− 0.081	0.392	114	− 0.176	**0.061**	114	0.084	0.376	114
DM other	− 0.031	0.756	104	0.023	0.808	114	− 0.042	0.661	114	− 0.162	0.085	114
DM bone only	− 0.030	0.760	104	− 0.155	0.100	114	0.053	0.573	114	0.095	0.314	114
Number of sites	0.058	0.557	104	0.089	0.344	114	− 0.028	0.764	114	− 0.075	0.425	114

^a^ Spearman’s 2-tailed test *P < 0.05, **P < 0.01, ***P < 0.001.

DM: distant metastasis.

*de novo* MBC: metastatic disease at initial breast cancer diagnosis.

To summarize, high amounts of CD15^+^MPO^+^ TANs or MPO^+^ cells at time of PT diagnosis enhanced the likelihood of having distant recurrent MBC (as opposed to *de novo* MBC), but high amounts of infiltrating CD15^+^MPO^+^ TANs or CD15^+^MPO^−^ TANs also decreased the likelihood of having bone and skin metastases, in contrast to MPO^+^ cells that increased the likelihood of lung metastasis.

### Presence of MPO^+^ TANs associate with total survival time from initial breast cancer diagnosis

**Fig. 3:**
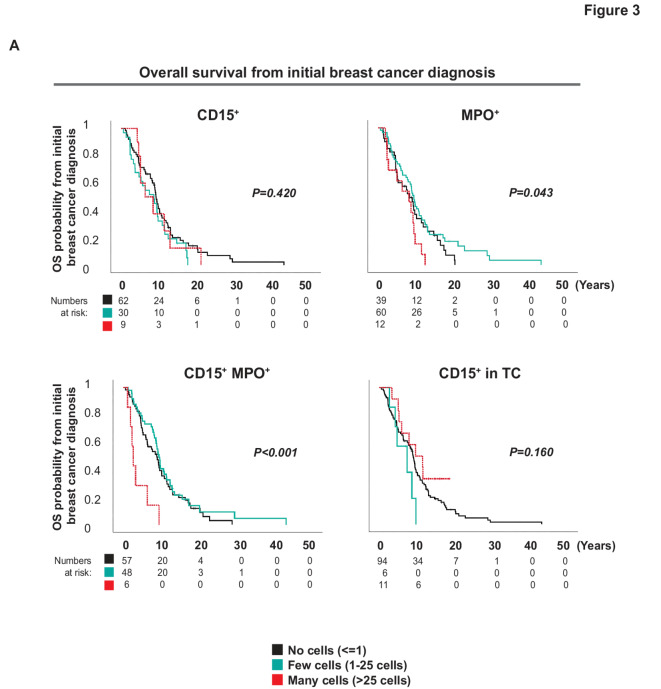
Presence of MPO^+^ TANs is associated with worse overall survival from initial breast cancer diagnosis in MBC patients. (**A**) Kaplan-Meier curves illustrating differences in Overall survival (OS) measured as time from initial breast cancer diagnosis according to CD15 and MPO expression in the PT from MBC patients. The cores were annotated for presence of cells expressing the markers according to grading no cells ( < = 1 cells; black), few cells (1–25 cells; turquoise) or many cells (> 25 cells; red dashed line). Log-rank *P*-value < 0.05 was considered significant.

To investigate the association of TANs and MPO-expressing cells on total survival time from initial breast cancer diagnosis, we next performed Kaplan Meier Log Rank tests, using the time scale OS as measured in time from initial breast cancer diagnosis (Fig. 3). Again, we could see that a higher number of CD15^−^MPO^+^ cells (MPO^+^; *P* = 0.043) and of MPO^+^ TANs in the PT (CD15^+^MPO^+^; *P* < 0.001) (Fig. 3) associated with worse survival. This was not seen for CD15^+^MPO^−^ (CD15^+^; non-activated or immature neutrophils; Fig. 3) (CD15^+^; *P* = 0.420), or CD15^+^ expression in malignant tumor cells (PFS CD15^+^ TC; *P* = 0.160; Fig. 3).

In summary, when evaluating OS time as measured from initial breast cancer diagnosis, high amounts of MPO^+^ cells and particularly of CD15^+^MPO^+^ TANs in PT, was associated with a worse prognosis.

## Discussion

To be able to target the devastating processes of cancer related to immune evasion, more knowledge is needed for every cancer type and group of patients. Today, the patients that benefit from immunotherapy increase as more research is conducted, but the comprehension regarding the involvement of innate immune cells on these therapies is still largely lacking. In breast cancer, the patient group that urgently need new targets for treatment is the metastatic breast cancer (MBC) group. Independent of breast cancer subtype, distant metastasis will lead to shorter life expectancy. It is therefore necessary to gain a better understanding of the innate immune system in these patients. In this study, we investigated the prognostic impact of presence of infiltrating neutrophils (TANs) of various activation stages, and the mediator myeloperoxidase (MPO) that is expressed in innate myeloid cells, in MBC patients. We found that MPO-expressing TANs and non-neutrophils (monocyte/macrophages) in the primary breast tumors (PT), were associated with a worse prognosis in MBC patients.

Neutrophils are necessary for fighting infections, but also have tumor-promoting functions. In neutropenic breast cancer patients that are routinely treated with G-CSF, more neutrophils and G-MDSCs will be generated^37,38^. Since G-MDSCs are characterized by a range of neutrophil maturation stages, MPO can guide in determining which maturation and activation stage of neutrophil biology that is important for pro- or anti-tumor functions when evaluating tumor associated neutrophils (TANs)^26^. To target the pro-tumor function of these cells will therefore be important for the future. MPO is a peroxidase that has received increasing interest as a potential therapy target in cancer patients^30^. Recently, inhibition of MPO in combination with ICI therapy was shown to be a promising strategy in in vivo melanoma models^39^ and pancreatic tumor models^40^. It is therefore of importance to reveal whether MPO has a prognostic impact in breast cancer patients, especially in the TNBC group that would benefit from ICI treatment the most, but also in MBC patients^41^. In this study, we show that indeed both MPO^+^ TANs (CD15^+^MPO^+^) and MPO^+^ non-neutrophils have a dismal impact on prognosis in MBC patients but did not correlate to any BC subtype. Importantly, only infiltration of CD15^+^MPO^+^ TANs was shown to be an independent prognostic factor after adjusted analyses, indicating that activated TANs in the PT have an unmistakable effect on prognosis in MBC patients. A possible mechanism behind our findings could be that only aggressive breast tumors with inflammatory mediator profiles attract TANs. In these tumors, MPO that clearly promotes progression of breast cancer in vivo^30^may be involved in promoting metastases also in human. However, a more likely scenario is that neutrophils may have opposite functions in early as compared to later tumor stages^16,42^since the different tumor immune microenvironments would affect neutrophil N1/N2 polarization differently.

We also showed that CD15^+^MPO^+^ TANs and MPO^+^ non-neutrophils correlated significantly to distant metastasis and shorter OS from time of initial breast cancer diagnosis, compared to tumors without infiltration of CD15^+^MPO^+^ TANs and MPO^+^ non-neutrophils. Since MPO^+^ TANs and MPO^+^ non-neutrophils did not correlate to any of the classical breast cancer subtypes, our findings may indicate that the PT tumors that have high numbers of MPO^+^ TANs and MPO^+^ non-neutrophils, might have a certain aggressive, inflammatory microenvironment. In this study, immature/non-activated TANs (CD15^+^MPO^−^) did not have a prognostic impact. This group of cells should include immature G-MDSCs with pro-tumoral functions. This was surprising to us and point in a direction where the mediator MPO, more likely to be found in activated neutrophils with NETs, is of more importance than the anti-inflammatory mediators produced by G-MDSCs^17,18,43^. Finally, the expression of CD15 in tumor cells (CD15^+^ TC) that we observed was both linked to DM to CNS and had an independent impact on MBC patients OS. It is possible that tumors cells expressing CD15 may represent a type of tumor cell with a signature that better suits chemoattraction to CNS than cancer cells without CD15 expression^44^. CNS metastases would most likely explain the reason to dismal prognosis in this group.

Our findings are in contradiction to a previous study regarding MPO as a biomarker for improved survival in breast cancer patients^32^. However, using a different cohort with primary breast cancer at earlier stages, we recently showed that a subpopulation of MPO^+^ neutrophils were associated with worse prognosis in breast cancer patients^33^. Although, as mentioned above, using the same cohort only MPO-expressing neutrophils were not prognostic^26^. The obvious difference between the studies are the patient cohorts, where in the present study all patients had MBC, but also the staining parameters of analyzing MPO in TANs or non-neutrophils. Neutrophils are notoriously difficult to characterize both using IHC and RNASeq analyses due to overlapping biomarkers with other myeloid cells and low mRNA yields in neutrophils, combined with the fact that the carbohydrate epitope CD15 does not have a fully representative gene in transcriptome analyses^45^. Using two immune cell markers to identify the MPO^+^ TANs or non-neutrophils (CD15 and MPO) as in our study, would theoretically detect most TANs, although not all. Also, the MPO^+^ non-neutrophils remain uncharacterized, however breast cancer public single cell RNA Seq data analyses indicate that they likely represent monocytes/macrophages^46^. An alternative interpretation is that TANs may have opposite mechanisms and impact in different tumors stages. In support of this, a study analysing neutrophils in tumor draining lymph nodes (TDLNs) of head and neck cancer patients^16^ showed that presence of neutrophils was associated with opposite prognosis if detected in TDLNs of early, as compared to later tumor stages, indicating a critical time dependent relation between tumor stage, neutrophils and lymphocyte activation. Finally, a limitation of our study is that the patient cohort used here is limited with only 114 patients. To our knowledge this is the first MBC cohort evaluated for MPO^+^ TANs to date and can therefore not be validated in another cohort at this time. Although power analysis was performed for the original cohort representing MBC in general, there is a pathological heterogeneity in the MBC cohort that limits the interpretation of the data at the detailed subtype level. The findings can be considered exploratory and need to be validated in a future larger cohort.

Whether MPO alone, or NETosis as such, mediates the mechanisms of action in relation to breast cancer metastasis and progression, needs further investigation. Additionally, more data is needed to confirm whether MPO is a possible target in humans, and this would in extension support therapy to be promptly evaluated in MBC patients. Indeed, our findings that also MPO^+^ non-neutrophils have a prognostic impact in this MBC patient cohort, may point in a direction of a direct molecular impact. Future analyses on the presence of MPO^+^ TANs or non-neutrophils in metastases of the MBC patients will hopefully lead to a better insight into whether they are promising therapeutical targets in MBC patients specifically. In summary, although they need to be validated in a larger cohort, the findings in our present study suggest that future clinical trials using immunotherapies targeting neutrophils and MPO should be focused on patients with particularly aggressive breast cancer and especially MBC patients.

## Methods

### Ethics statement

All patient sample collections were approved by the local Regional Ethical Committee in Lund, Sweden. A written informed consent was obtained from patients with metastatic breast cancer and ethical approval was obtained from the Regional Ethics Committee in Lund, Sweden (Dnr 2010/135) and conducted in accordance with the Declaration of Helsinki.

### Patient samples

The cohort consists of 114 primary breast tumor samples from patients with newly diagnosed metastatic breast cancer (MBC). Patients were included in a prospective observational trial with long-term follow up (ClinicalTrials.gov NCT01322893) 2011–2016 at Skåne University Hospital and Halmstad county Hospital, Sweden. The inclusion criteria were age > = 18 years, MBC, predicted life expectancy more than two months and a performance status score 0–2 on the Eastern Cooperative Oncology Group (ECOG) scale. Exclusion criteria included previous systemic therapy for MBC and unrelated malignant disease during the last 5 years. Patients received systemic therapy in line with national clinical guidelines and the median follow up time was 97 months (75–123 months). Progression was defined by modified RECIST criteria based on collected radiological and clinical data. The patient characteristics and clinical variables have previously been published^34–36^ and a summary in comparison to the original cohort is shown in Supplementary Table 1.

### Immunohistochemistry (IHC)

We mounted 4-µm thick sections of paraffin-embedded tumor tissue arranged in a TMA onto glass slides. This was followed by deparaffinization and antigen retrieval using the PT-link system (Agilent, Santa Clara, CA) and staining using Autostainer Plus (Agilent) and EnVisionFlex High pH kit (Agilent). Antibodies used were anti-CD15 (human; clone Ab754; dilution 1:100; 30 min HIER pH9; Abcam) and anti-MPO (human; clone A0398; dilution 1:1000; 30 min HIER pH9; Agilent), with secondary antibodies conjugated to DAB (CD15, brown) and magenta (MPO, pink) using Envision Flex and Envision Flex HRP Magenta Substrate chromogen from DAKO (Agilent). Omnis Sulfuric acid 0,3 M was used as blocker between antibody incubations. CD15^+^MPO^−^ (CD15^+^), CD15^−^MPO^+^ (MPO^+^) and CD15^+^/MPO^+^ expression in ICs, or CD15^+^ expression in tumor cells (CD15^+^ TC), were annotated as the absence (no cells expressing the indicated markers; <=1 cell), or presence of low infiltration (few cells; 1–25 cells) or high infiltration (many cells; >25 cells) by two independent assessors and confirmed by supervising assessor (Fig. 1A). In 10 tumor cores the CD15 staining could not be confidently identified as belonging to an immune cell (IC) or other cell type and hence not scored, therefore CD15^+^MPO^−^ cells were only annotated in 104 tumors. All histological sections were counterstained with H&E. Only cores with cancer cells present were annotated.

### Statistical analysis

Statistical power calculations were performed for the original patient cohort as previously described^36^. Time from study inclusion to progression (PFS) or death from any cause (OS) was calculated. For calculation of total survival time from initial breast cancer diagnosis, MFI was added to OS for each patient. If an outcome was not reached, time variables were censored at the last follow up.

IBM SPSS Statistics v 29.0.2.0 (SPSS Inc.) and Graph Pad Prism 9 software were used for statistical analyses. Correlations to clinicopathological variables were analyzed using bivariate Spearman´s 2-tailed correlation analyses test as indicated in table legends. Spearman´s test was chosen due to non-parametric or non-continuous data in Tables 1 and 2. All *P* values presented are two-sided. Kaplan-Meier analyses and log rank tests were used to illustrate differences in progression free survival (PFS) and overall survival (OS) according to CD15 and MPO expression. Cox regression models were used for estimation of hazard ratios (HR) according to CD15 and MPO expression in multivariable analysis adjusted for other prognostic factors. Cox regression was chosen as statistical test since it predicts the variables that significantly affects the survival based on multiple assumptions and is commonly used for survival analyses in epidemiological and clinical research. No correction for multiple testing was performed due to the exploratory nature of the study.

## Electronic supplementary material

Below is the link to the electronic supplementary material.

Supplementary Material 1

Supplementary Material 2

## Data Availability

All datasets generated in the course of the current study are presented in the main text and the Supplementary Information available online.

## Abbreviations

AIRE: Autoimmune regulator
DM: Distant metastasis
ER: Estrogen receptor
ECOG: Eastern cooperative oncology group
G-MDSC or PMN-MDSC: Granulocytic myeloid derived suppressor cells
HER2: Human epidermal growth factor receptor 2
HR: Hazard ratios
H&E: Hematoxylin and Eosin
IC: Immune cells
ICI: Immune checkpoint inhibitors
IHC: Immunohistochemistry
MBC: Metastatic breast cancer
MDCS: Myeloid derived suppressor cells
MFI: Metastasis-free interval
MPO: Myeloperoxidase
MV: Multivariate
NETs: Neutrophil extracellular traps
NHG: Nottingham grade
OS: Overall survival
PFS: Progression free survival
PR: Progesterone receptor
PT: Primary tumor
ROS: Reactive oxygen species
TAMs: Tumor associated macrophages
TANs: Tumor associated neutrophils
TDLN: Tumor draining lymph nodes
TC: Tumor cells
TILs: Tumor infiltrating lymphocytes
TME: Tumor microenvironment
TNBC: Triple negative breast cancer

## Declarations

### Data and materials availability

All datasets generated in the course of the current study are presented in the main text and the Supplementary Information available online.
