# Supplementary material for: Myeloperoxidase expressing tumor associated neutrophils are associated with worse prognosis in metastatic breast cancer patients
Source: Sci Rep. 2025 Jul 12;15:25270. doi: 10.1038/s41598-025-08854-x (PMC12255714; doi:10.1038/s41598-025-08854-x)
Supplement: Supplementary file 1 — Supplementary Material 1 [file 41598_2025_8854_MOESM1_ESM.docx]

**Supplementary Table 1. Patient and tumor characteristics of the CD15/MPO cohort compared to the original cohort**.

| ***Variable*** | ***CD15/MPO cohort***  ***Total n =114*** | ***Whole original cohort***  ***Total n=156*** |
| --- | --- | --- |
| **Age at MBC diagnosis (years)**  < 65  ≥ 65 | 56 (49 %)  58 (51 %) | 75 (48%)  81 (52%) |
| **ECOG at MBC diagnosis**  0  1  2  Unknown | 67 (59 %)  31(27 %)  15 (13 %)  1 | 91 (61 %)  37 (25 %)  22 (15 %)  6 |
| **PT NHG**  I  II  III  Unknown | 8 (9 %)  45 (50 %)  37 (41 %)  24 | 13 (11 %)  65 (52 %)  46 (37 %)  32 |
| **PT tumor size**  T1  T2  T3  T4  Unknown | 39 (36%)  41 (38%)  14 (13 %)  14 (13%)  6 | 57 (39 %)  51 (35 %)  20 (13 %)  19 (13 %)  9 |
| **PT node status**  Neg  Pos  Unknown | 31 (31%)  68 (69%)  15 | 44 (32 %)  92 (68 %)  20 |
| **PT ER status**  Neg  Pos  Unknown | 19 (17%)  91 (83%)  4 | 24 (16%)  123 (84%)  9 |
| **PT HER2 status**  Neg  Pos  Unknown | 80 (86%)  13 (14%)  21 | 109 (86%)  18 (14%)  29 |
| **PT PAM50 subtype**  Luminal A  Luminal B  HER2E  Basal-like  Unknown | 45 (40%)  39 (35%)  15 (13%)  14 (12%)  1 | 47 (38%)  45 (37%)  15 (12%)  16 (13%)  33 |
| **Subtype (IHC) metastases first**  Estrogen receptor-positive (ER^+^, HER2^-^)  HER2-positive  TNBC  Unknown | 78 (72%)  14 (13%)  17 (16%)  5 | 105 (69.5 %)  20 (13.2 %)  26 (17.2 %)  5 |
| **Metastasis free interval (MFI)**  0 years (*de novo* MBC)  > 0 but ≤ 3 years  > 3 years | 23 (20%)  20 (18%)  71 (62%) | 31 (19.9 %)  28 (17.9 %)  97 (62.2%) |
| **Metastatic sites, *n***  < 3  ≥ 3 | 75 (66%)  39 (34%) | 109 (69.9 %)  47 (30.1 %) |
| **Metastatic localization**  Visceral  No  Yes | 46 (40%)  68 (60%) | 65 (41.7 %)  91 (58.3 %) |
| **CTC at BL**  < 5  ≥ 5  Unknown | 55 (48%)  59 (52%) | 73 (48.0 %)  79 (52.0 %)  4 |
| **1^st^ line systemic therapy**  Chemotherapy  Endocrine therapy  HER2-targeted therapy  Unknown | 54 (50%)  43 (40%)  10 (9%)  7 | 71 (49%)  58 (40%)  15 (10%)  12 |
