# Supplementary material for: Myeloperoxidase expressing tumor associated neutrophils are associated with worse prognosis in metastatic breast cancer patients
Source: Sci Rep. 2025 Jul 12;15:25270. doi: 10.1038/s41598-025-08854-x (PMC12255714; doi:10.1038/s41598-025-08854-x)
Supplement: Supplementary file 2 — Supplementary Material 2 [file 41598_2025_8854_MOESM2_ESM.docx]

**Supplementary Table 2. Correlations^a^ between the annotated immune infiltrates in the primary breast tumor (PT) of metastatic breast cancer (MBC) patients.**

| **Immune infiltrate** | **CD15^+^** | **MPO^+^** | **CD15^+^MPO^+^** | **CD15^+^ TC** |
| --- | --- | --- | --- | --- |
|  | Correlation *P* value N  Coefficient (2-tailed) | Correlation *P* value N  Coefficient (2-tailed) | Correlation *P* value N  Coefficient (2-tailed) | Correlation *P* value N  Coefficient (2-tailed) |
| **CD15^+^**  **MPO^+^**  **CD15^+^MPO^+^**  **CD15^+^ TC** | 1.000 - 104  0.079 0.434 104  -0.014 0.891 104  **0.365 <0.001*** 104** | 0.079 0.434 104  1.000 - 114  **0.486 <0.001*** 114**  0.019 0.840 114 | -0.014 0.891 104  **0.486 <0.001*** 114**  1.000 - 114  -0.045 0.631 114 | **0.365 <0.001*** 104**  0.019 0.840 114  -0.045 0.631 114  1.000 - 114 |

^a^ Spearman’s 2-tailed test * *P*<0.05, ** *P*<0.01, *** *P* <0.001.
